# Type I interferons regulate eomesodermin expression and the development of unconventional memory CD8^+^ T cells

**DOI:** 10.1038/ncomms8089

**Published:** 2015-05-08

**Authors:** Valérie Martinet, Sandrine Tonon, David Torres, Abdulkader Azouz, Muriel Nguyen, Arnaud Kohler, Véronique Flamand, Chai-An Mao, William H. Klein, Oberdan Leo, Stanislas Goriely

**Affiliations:** 1 WELBIO and Institute for Medical Immunology (IMI), Université Libre de Bruxelles, Gosselies 6041, Belgium; 2 Department of Systems Biology, University of Texas, MD Anderson Cancer Center, Houston, Texas 77030, USA

## Abstract

CD8^+^ T-cell memory phenotype and function are acquired after antigen-driven activation. Memory-like cells may also arise in absence of antigenic exposure in the thymus or in the periphery. Eomesodermin (Eomes) is a key transcription factor for the development of these unconventional memory cells. Herein, we show that type I interferon signalling in CD8^+^ T cells directly activates *Eomes* gene expression. Consistent with this observation, the phenotype, function and age-dependent expansion of ‘virtual memory' CD8^+^ T cells are strongly affected in absence of type I interferon signalling. In addition, type I interferons induce a sustained expansion of ‘virtual memory' CD8^+^ T cells in an Eomes-dependent fashion. We further show that the development of ‘innate thymic' CD8^+^ T cells is dependent on the same pathway. In conclusion, we demonstrate that type I interferon signalling in CD8^+^ T cells drives *Eomes* expression and thereby regulates the function and homeostasis of memory-like CD8^+^ T cells.

## Abstract (web summary)

Eomesodermin is the key transcription factor for the development of ‘virtual memory' T cells that develop in the absence of antigen-driven activation. Here the authors show that type I interferons directly activate eomesodermin and contribute to the homeostasis of virtual memory CD8^+^ T cells.

## Graphical abstract

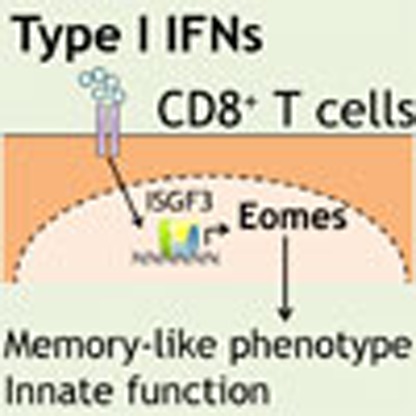

CD8^+^ T cells are important effectors of the immune response against tumours, viruses and other intracellular pathogens. During infection or vaccination, CD8^+^ T cells undergo antigen-specific activation and expansion to give rise to cellular progeny, acquiring effector functions for pathogen clearance. The pool of activated CD8^+^ T cells then undergoes a contraction phase, leaving behind a small fraction of memory cells that contributes to antigen-specific life-long protection^1^,^2^. In absence of antigen exposure, CD8^+^ T cells may also acquire a memory phenotype in the thymus (‘innate-like' CD8^+^ T cells)^3^,^4^ or in the periphery (‘virtual memory' (VM) cells)^5^,^6^. Recent evidences indicate that conventional and unconventional memory CD8^+^ T-cell subsets promptly secrete large amounts of cytokines in response to inflammatory cues in the context of infection^7^,^8^. This non-cognate activation of memory CD8^+^ T cells that leads to rapid interferon (IFN)γ production and acquisition of cytolytic functions contributes to the first line of defence and favours a Th1-prone environment^6^,^7^,^9^,^10^,^11^.

The transcriptional networks implicated in the alternative differentiation of memory-phenotype CD8^+^ T cells are poorly understood. In these subpopulations, Eomesodermin (Eomes), a transcription factor closely related to T-bet, appears to play a central role in the acquisition of memory phenotype and function^12^,^13^,^14^. In conventional memory cells, Eomes favours the development of central memory cells (T_CM_) characterized by longer survival and an important potential for homeostatic proliferation^15^,^16^. However, in the context of chronic viral infection, Eomes is also important for the terminal differentiation of virus-specific CD8^+^ T cells in response to persisting antigen^17^. In different mice models that give rise to innate-like CD8^+^ T cells, interleukin (IL)-4-dependent Eomes induction within CD8 single-positive (SP) thymocytes is required for their differentiation^12^,^14^,^18^,^19^. The development of VM CD8^+^ T cells in the periphery also relies on high Eomes expression that mediates CD122 expression and responsiveness to IL-15 trans-presentation by CD8α dendritic cells^13^. Despite the important role of Eomes in these contexts, the signalling pathways responsible for its sustained expression in memory CD8^+^ T cells are still ill-defined.

Type I IFNs display important direct and indirect immunomodulatory effects on CD8^+^ T cells^20^,^21^. They promote the expression of specific cytokines by antigen-presenting cells (APCs) such as IL-15 or IL-27, which play a critical role in CD8^+^ T-cell activation or differentiation^22^,^23^,^24^,^25^. Similar to IL-12, they act as a ‘third signal' that promotes full activation, proliferation and survival of CD8^+^ T cells activated by T cell receptor and costimulatory molecules^21^,^26^. In contrast, several studies showed that type I IFNs generally inhibit CD8^+^ T-cell proliferation by increasing their sensitivity to apoptosis^27^,^28^,^29^. These mediators also induce the rapid acquisition of effector functions in absence of antigenic stimulation both in naive and memory cells^30^,^31^. Type I IFNs activate multiple signal transducer and activator of transcription (STAT) molecules, including STAT1, STAT3 homo/heterodimers and the IFN-stimulated gene factor 3 (ISGF3) complex composed of STAT1, STAT2 and IFN regulatory factor (IRF) 9 (ref. 21).

In the present work, we demonstrate that type I IFNs induce direct *Eomes* gene expression through activation of the ISGF3 complex within CD8^+^ T cells. We further show that this pathway contributes to the homeostasis and innate functions of memory-like CD8^+^ T cells both in the periphery and in the thymus.

## Results

### Reduced pool of VM CD8^+^ T cells in IFNAR^−/−^ mice

**Figure 1:**
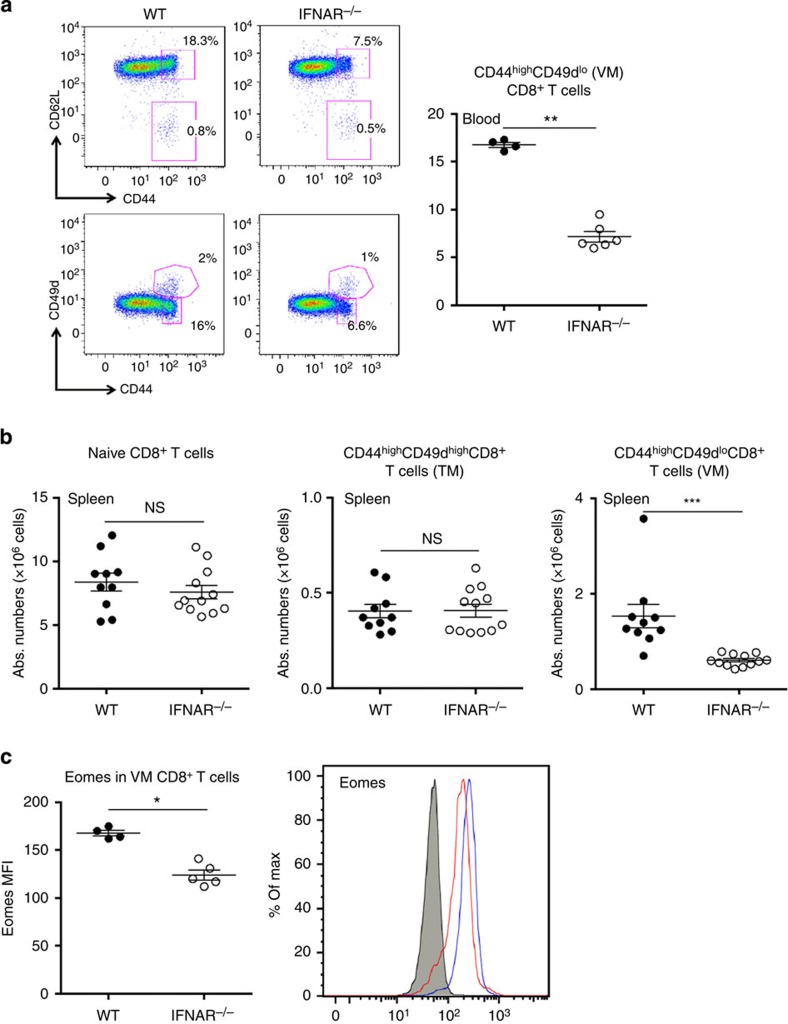
The pool of virtual memory CD8^+^ T cells is decreased in IFNAR^−/−^ mice. (**a**) Total circulating CD8^+^ T cells were analysed *ex vivo* for the frequency of memory CD44^+^CD62L^+^CD8^+^ T cells, virtual memory CD44^+^CD49d^lo^ CD8^+^ T cells (VM) and true memory CD44^+^CD49d^hi^ CD8^+^ T cells (TM) in WT and IFNAR^−/−^ mice. (**b**) Absolute numbers of naive, VM and TM CD8^+^ T cells in the spleens of WT and IFNAR^−/−^ mice. (**c**) Mean fluorescence intensity (MFI) of Eomes in VM CD8^+^ T cells. Representative histograms of Eomes expression in WT (blue line) and IFNAR^−/−^ (red line) VM CD8^+^ T cells are shown. As a control, we included staining in CD8^+^ T cells from Eomes^fl/fl^CD4^cre^ mice (plain histogram). *P<0.05, **P<0.01 and ***P<0.001, NS: not significant (non-parametric Mann–Whitney). In graphs, each dot represents an individual mouse and bars represent mean±s.e.m.

Type I IFNs are known to regulate immune cell homeostasis through their ability to affect cellular proliferation and survival^20^. In an initial set of experiments, we analysed the relative frequency of CD8^+^ T-cell subpopulations in naive mice lacking type I IFN receptor (IFNAR^−/−^ mice). We observed that the pool of memory CD44^+^CD62L^+^CD8^+^ T cells was reduced in these mice as compared with age-matched wild-type (WT) animals (Fig. 1a). Recent data indicate that a significant proportion of memory CD8^+^ T cells in the periphery of naive animals are not antigen-experienced ‘true memory' (TM) cells but so-called ‘VM' cells that express low levels of CD49d, an integrin alpha subunit^5^,^13^,^32^. We observed that the reduced proportion of memory cells in IFNAR^−/−^ mice was due to lower frequency and absolute counts of VM cells (Fig. 1a,b). The pool of TM cells was not consistently altered in IFNAR^−/−^ mice (Fig. 1b). As Eomes plays a critical role in the development of this particular memory subset^13^,^33^, we evaluated its expression in VM cells from both strains. We observed a significant decrease in Eomes expression in absence of type I IFN signalling (Fig. 1c).

### Type I IFNs directly activate *Eomes* expression in CD8^+^ T cells

**Figure 2:**
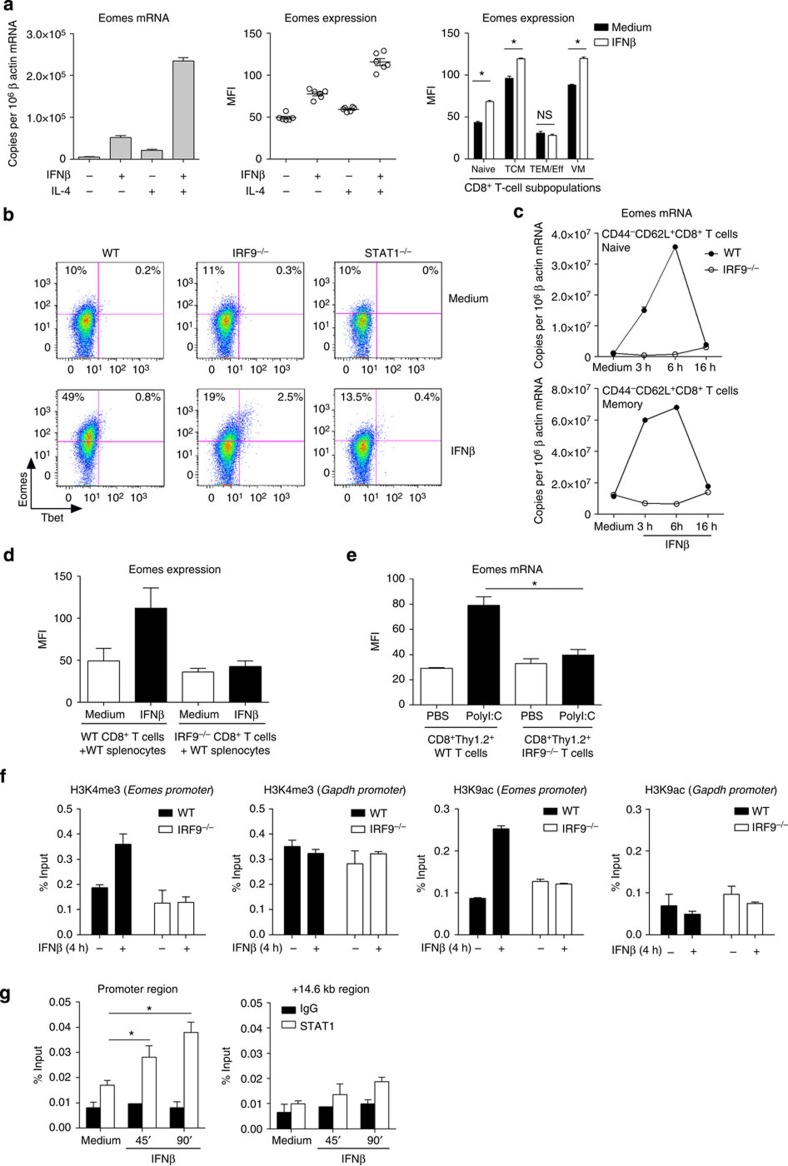
Type I IFNs directly activate *Eomes* gene expression in CD8^+^ T cells. (**a**) Purified CD8^+^ T cells were incubated in medium alone or stimulated with rIFNβ (100 U ml^−1^) and/or rIL-4 (20 ng ml^−1^). After 6 h of stimulation, Eomes mRNA levels were quantified and normalized against β-actin mRNA levels. Histograms represent mean±s.e.m. of triplicates (representative of three independent experiments). Eomes expression (mean fluorescence intensity (MFI)) in total or the indicated subpopulation of CD8^+^ T cells was assessed by flow cytometry after 16 h of stimulation. (**b**) Splenocytes from WT, IRF9^−/−^ or STAT1^−/−^ mice were cultured for 16 h with or without rIFNβ (100 U ml^−1^) in presence of rIL-2 (100 U ml^−1^). Eomes expression in total CD8^+^ T cells was assessed by flow cytometry. (**c**) Naive CD44^−^CD8^+^ T cells and CD44^+^CD62L^+^ memory CD8^+^ T cells were sorted and cultured for the indicated time with or without rIFNβ (100 U ml^−1^). Eomes mRNA levels were normalized against β-actin mRNA levels (experiment performed in triplicates). (**d**) WT or IRF9^−/−^ Thy1.2^+^ CD8^+^ T cells were cultured with WT Thy1.1^+^ splenocytes for 16 h with or without rIFNβ (100 U ml^−1^). Eomes expression in WT or IRF9^−/−^ Thy1.2^+^ CD8^+^ T cells is shown. Mean±s.e.m. of two independent experiments is shown. (**e**) WT or IRF9^−/−^ Thy1.2^+^ CD8^+^ T cells were adoptively transferred i.v. into WT Thy1.1^+^ mice 24 h before PBS or polyI:C (200 μg per mouse) injection (intraperitoneally). Mice were killed 16 h post injection. Eomes expression within the Thy1.2^+^ CD8^+^ T-cell population was analysed by flow cytometry. Histograms represent mean±s.e.m. (five mice per group). (**f**) Total CD8^+^ T cells from WT and IRF9^−/−^ mice were stimulated for 4 h with IFNβ or medium alone. H3K4me3 and H3K9Ac modifications in the proximal promoter region of the *Eomes* or *Gapdh* loci were quantified by quantitative (q)PCR after chromatin immunoprecipitation (ChIP) experiments (normalization against input controls). Values are expressed as mean±s.e.m. of duplicates and are representative of two independent experiments. (**g**) Total CD8^+^ T cells from WT mice were incubated with rIFNβ or medium alone for the indicated time. STAT1 recruitment to the proximal region or to a control region located +14.6 kb downstream of the transcriptional start site of the *Eomes* gene was quantified by qPCR after ChIP experiments (normalization against input controls). Values represent mean±s.e.m. of three independent experiments. *P<0.05 (non-parametric Mann–Whitney).

On the basis of these initial results, we postulated that type I IFN signalling could play a role in the regulation of VM CD8^+^ T-cell homeostasis possibly by regulating Eomes expression. To test this hypothesis, we incubated isolated CD8^+^ T cells with recombinant IFNβ. As a positive control, we used rIL-4 that activates Eomes expression in a STAT6-dependent fashion^18^,^34^,^35^. In these experimental conditions, rIFNβ alone or in combination with rIL-4 was a potent inducer of Eomes expression both at the messenger RNA (mRNA) and the protein levels (Fig. 2a). This effect was observed both in naive and CD44^+^CD62L^+^CD49d^low^ (‘VM') or CD49d^high^ (‘central memory') subsets. As previously shown^15^, Eomes expression in CD44^+^CD62L^−^ effector cells was lower than in the other memory subsets and was not upregulated in response to rIFNβ (Fig. 2a).

Type I IFNs signal through the formation of the ISGF3 complex (STAT1/2/IRF9). We assessed the specific role of this pathway by using CD8^+^ T cells from IRF9^−/−^ and STAT1^−/−^ mice. Induction of Eomes by rIFNβ was found to be IRF9- and STAT1-dependent (Fig. 2b). T-bet expression was not significantly modulated in these conditions, indicating that type I IFN signalling differentially regulates these two related transcription factors. We further confirmed the role of IRF9 in type I IFN-induced Eomes mRNA upregulation both in naive and CD44^+^CD62L^+^ memory subsets (Fig. 2c).

Previous studies indicated that type I IFNs prime CD8^+^ T-cell effector function by indirectly targeting APCs^31^. To assess a possible CD8^+^ T-cell extrinsic role of IRF9 signalling on Eomes expression, we performed co-culture *in vitro* experiments. For this purpose, Thy1.2^+^ CD8^+^ T cells purified from WT or IRF9^−/−^ mice were cultured with congenic WT Thy1.1^+^ splenocytes for 16 h with or without rIFNβ. Eomes expression was analysed by flow cytometry (Fig. 2d). We observed that IRF9^−/−^ CD8^+^ T cells exhibited impaired expression of Eomes in response to rIFNβ even in presence of WT splenocytes. Next, we analysed Eomes expression upon *in vivo* induction of type I IFNs by injection of polyI:C. To study the CD8^+^ T-cell intrinsic role of IRF9, we performed adoptive transfers of WT or IRF9^−/−^ Thy1.2^+^ CD8^+^ T cells into congenic Thy1.1^+^ WT mice before polyI:C injection. Sixteen hours after injection, Eomes expression was analysed by flow cytometry in spleen cells. We observed a clear upregulation of Eomes upon polyI:C injection in WT but not in IRF9-deficient CD8^+^ T cells (Fig. 2e). These experiments indicate that type I IFN signalling within CD8^+^ T cells leads to the activation of *Eomes* both *in vitro* and *in vivo*.

Next, we looked at classical histone modifications in the proximal promoter region of *Eomes* (Fig. 2f). We found an IRF9-dependent increase in H3K4me3 and H3K9Ac in response to rIFNβ, highly suggestive of transcriptional activation. We identified a putative IFN-stimulated response element site located 150 base pairs upstream of the transcriptional start site. We observed direct recruitment of STAT1 to this region in chromatin immunoprecipitation experiments (Fig. 2g). In contrast, STAT1 recruitment to a control region located downstream of the gene body did not increase above the background levels. Taken together, these experiments indicate that type I IFNs directly activate *Eomes* gene expression in CD8^+^ T cells in a STAT1/IRF9-dependent fashion.

### IRF9 regulates the homeostasis of memory CD8^+^ T cells

**Figure 3:**
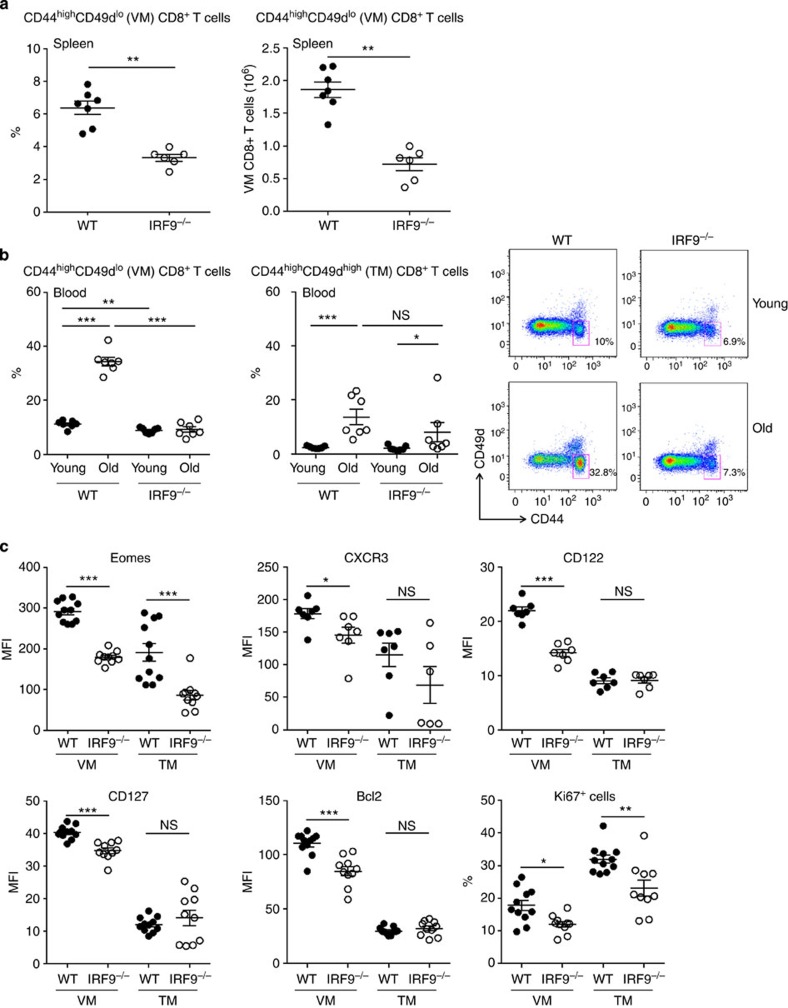
IRF9 regulates the homeostasis of memory CD8^+^ T cells. (**a**) Frequency and absolute numbers of splenic VM CD8^+^ T cells in 6–8-week-old WT and IRF9^−/−^ mice (representative groups of three independent experiments). (**b**) Frequency of circulating CD44^+^CD49d^lo^ VM and CD44^+^CD49d^hi^ TM CD8^+^ T cells in young (6–10 weeks) and aged (>10 month) WT and IRF9^−/−^ mice. Representative dot plots (out of at least seven mice) and graphs are shown. (**c**) Expression of Eomes, CD122, CD62L, CxCR3, CD127, Bcl2 (mean fluorescence intensity (MFI)) and Ki67 (%) among CD44^+^CD49d^lo^ and CD44^+^CD49d^hi^ CD8^+^ T cells from age-matched WT and IRF9^−/−^ old mice. For all graphs, each dot represents an individual mouse. Bars represent mean±s.e.m. *P<0.05, **P<0.01 and ***P<0.001, NS: not significant.

Our results indicate that IRF9 is critical for type I IFN-induced Eomes expression. We therefore analysed CD8^+^ T-cell memory subsets in IRF9^−/−^ mice. As observed in IFNAR^−/−^ mice (Fig. 1), we found that the frequency and absolute counts of VM cells was significantly lower in naive IRF9^−/−^ mice as compared with WT animals (Fig. 3a).

In agreement with recent reports^32^,^36^, we observed an important expansion of circulating memory CD44^+^CD49d^lo^ CD8^+^ T cells in old (>10 months) WT mice as compared with younger (6–8 weeks) mice (Fig. 3b). Interestingly, the age-associated expansion of the VM compartment was totally abrogated in absence of IRF9 (Fig. 3b). In contrast, the frequency and age-dependent expansion of CD44^+^CD49d^high^ TM CD8^+^ T cells was not affected by the lack of IRF9 (Fig. 3b).

Eomes is strongly implicated in the development of VM cells^13^,^14^. As compared with antigen-experienced (CD49d^high^) memory cells, Eomes was found to be highly expressed in circulating VM cells from WT old animals (Fig. 3c). Its expression was significantly decreased in age-matched IRF9^−/−^ animals (Fig. 3c) both in VM and TM CD8^+^ T cells. VM cells are characterized by high levels of CXCR3 and CD122, which are direct or indirect targets of Eomes^37^,^38^,^39^,^40^. We found that expression of these two surface molecules was significantly decreased in IRF9^−/−^ VM cells (Fig. 3c). We observed that CD127 was highly expressed in VM as compared with TM CD8^+^ T cells. CD127 expression was also significantly decreased in VM cells from IRF9^−/−^ old animals (Fig. 3c).

To gain further insight into the mechanisms responsible for decreased frequency of VM cells in IRF9^−/−^ mice, we analysed Ki67 and Bcl2 expression within these subsets. Interestingly, we found that the expression of these molecules also strongly differs between VM and TM CD8^+^ T cells. While Bcl2 was highly expressed in VM CD8^+^ T cells, Ki67 levels were higher in TM cells. We observed that expression of both molecules was reduced in IRF9^−/−^ cells, indicating lower proliferation rate and an increase in their sensitivity to apoptosis (Fig. 3c). Taken together, these results indicate that VM cells display a unique phenotype that favours their responsiveness towards homeostatic cytokines such as IL-7 and IL-15. Our results further suggest that IRF9-dependent signals are critical for their general fitness.

### Type I IFNs regulate innate IFNγ production by CD8^+^ T cells

**Figure 4:**
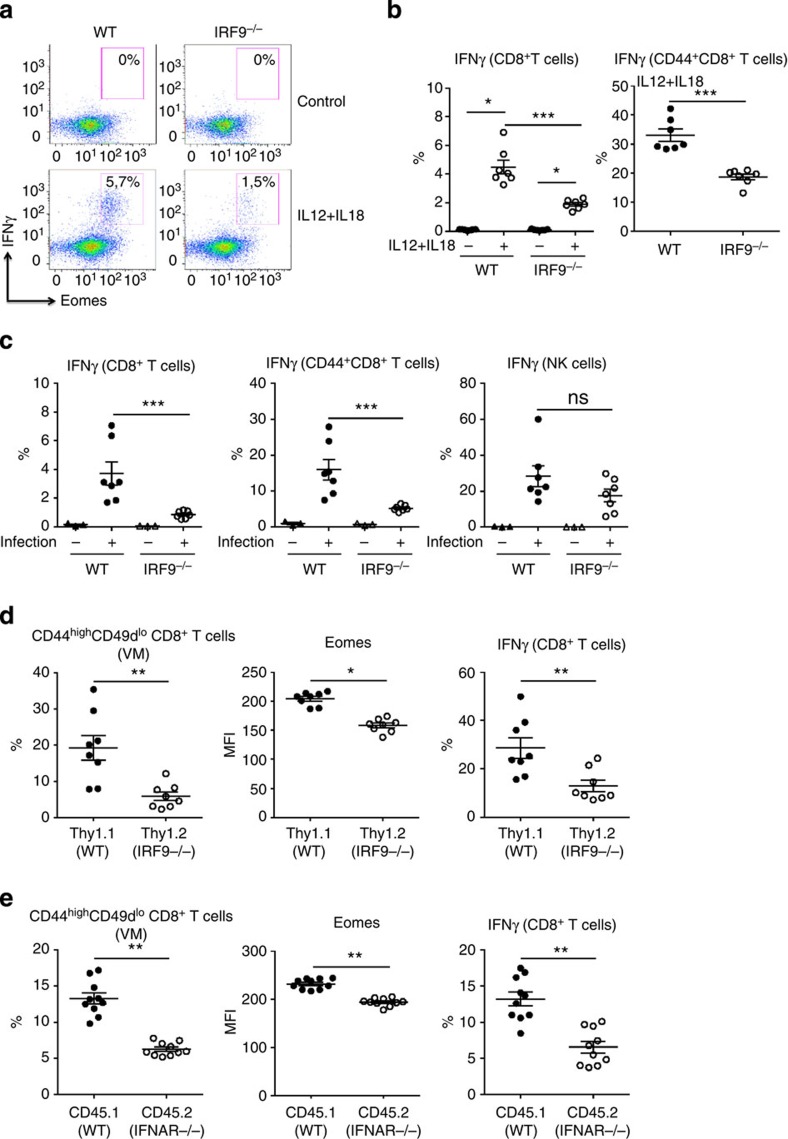
Type I IFNs regulate innate IFNγ production by CD8^+^ T cells. (**a**,**b**) Splenocytes from WT and IRF9^−/−^ mice were cultured for 16 h with or without IL-12+IL-18. Brefeldin A (5 μg ml^−1^) was added for the last 3 h of culture. (**a**) Dot plots of IFNγ and Eomes expression among total CD8^+^ T cells from WT and IRF9^−/−^ mice are illustrated. (**b**) Frequency of IFNγ^+^ cells among total and memory CD44^+^ CD8^+^ T cells in WT and IRF9^−/−^ mice. Data shown are representative of at least three independent experiments. (**c**) WT and IRF9^−/−^ mice were infected with Listeria *monocytogenes* 1 day before killing. Splenocytes were isolated and cultured for 4 h in presence of Brefeldin A before intranuclear staining and analysis by flow cytometry. Total CD8^+^ T cells, CD44^+^CD8^+^ T cells and NK cells were analysed for IFNγ expression. (**d**,**e**) Mixed bone marrow chimera experiments. WT Thy1.1^+^ and IRF9^−/−^ Thy1.2^+^ (**d**) or WT CD45.1 and IFNAR^−/−^ CD45.2 (**e**) myeloid progenitors were transferred into the irradiated RAG2^−/−^ host. After 3 months, the frequency of CD44^+^CD49d^lo^ CD8^+^ T cells among WT and IRF9^−/−^ or IFNAR^−/−^ CD8^+^ T cells was analysed by flow cytometry. Eomes expression levels are represented among WT and IRF9^−/−^ or IFNAR^−/−^ VM CD8^+^ T cells. Right panel: splenocytes from the chimeras were cultured for 16 h with IL-12+IL-18. Brefeldin A (5 μg ml^−1^) was added for the last 3 h of culture. The frequency of IFNγ^+^ cells among WT and IRF9^−/−^ or IFNAR^−/−^ total CD8^+^ T cells are shown. For all graphs, each dot represents an individual mouse. Bars represent mean±s.e.m. *P<0.05, **P<0.01 and ***P<0.001, NS: not significant (non-parametric Mann–Whitney or paired Wilcoxon test for mixed bone marrow chimera experiments).

To determine the innate functions of these memory CD8^+^ T cells in steady-state conditions, we stimulated splenocytes from naive 6–8-week-old WT and IRF9^−/−^ animals with rIL12+rIL18, a combination known to trigger rapid IFNγ production by memory CD8^+^ T cells in a non-cognate fashion^9^,^22^. IFNγ production in these conditions was restricted to Eomes^high^ memory CD8^+^ T cells (Fig. 4a) and was found to be decreased in IRF9^−/−^ CD8^+^ T cells (Fig. 4b). This was not only the consequence of the reduced memory pool, as IRF9^−/−^ CD44^+^ CD8^+^ T cells displayed lower IFNγ production as compared with their WT counterparts (Fig. 4b).

To further assess the functional implications of our observations in the acquisition of innate effector functions in the context of acute *Listeria monocytogenes* (LM) infection, we analysed IFNγ expression in WT and IRF9^−/−^ mice 16 h post infection. *Listeria*-elicited IFNγ production by CD8^+^ T cells was strongly decreased in IRF9^−/−^ mice (Fig. 4c). In contrast, IFNγ production by natural killer (NK) cells was not affected in *Listeria*-infected IRF9^−/−^ mice, indicating that the inflammatory cues that induce IFNγ production were globally maintained in these mice (Fig. 4c).

To define whether IRF9 acts in a T-cell intrinsic fashion, we performed mixed bone marrow chimera by transferring WT Thy1.1^+^ and IRF9^−/−^ Thy1.2^+^ myeloid progenitors into an irradiated RAG2^−/−^ host. Ten weeks after reconstitution, we observed that the proportion of memory CD8^+^ T cells was reduced among the IRF9^−/−^ CD8^+^ T cells as compared with WT CD8^+^ T cells. This was reflected by a decrease in the VM subset and reduced Eomes expression within memory cells, demonstrating the T-cell intrinsic role of IRF9 in our observations (Fig. 4d). Upon *ex vivo* rIL-12+rIL-18 stimulation, we observed that IRF9^−/−^ cells produced lower IFNγ levels. As IRF9 could play a T-cell intrinsic role independently of type I IFN signalling, we performed similar mixed bone marrow chimera with WT and IFNAR^−/−^ cells (Fig. 4e). We confirmed our findings in this experimental setting, showing that type I IFN signalling within CD8^+^ T cells contributes to the homeostasis of ‘VM' CD8^+^ T cells and their innate functions.

### PolyI:C injection increases the pool of VM CD8^+^ T cells

**Figure 5:**
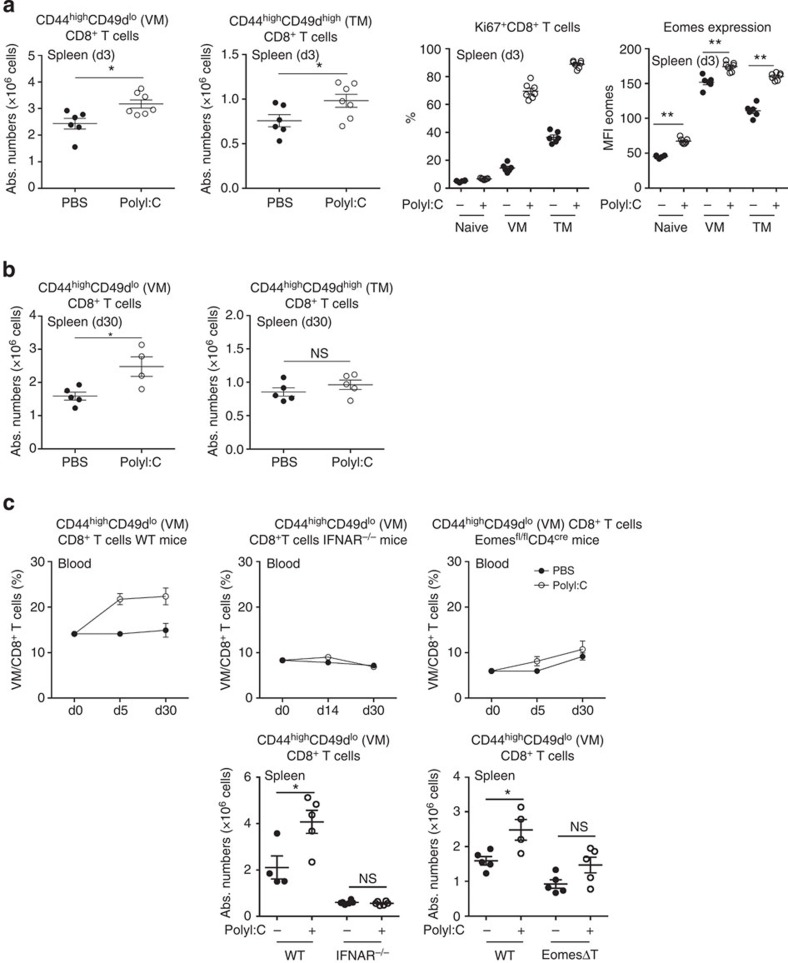
PolyI:C injection increases the pool of VM CD8^+^ T cells. (**a–c**) Mice were injected with 200 μg of polyI:C intraperitoneally (**a**) Three days after injection, mice were analysed for the absolute numbers of VM and TM CD8^+^ T-cell subsets in the spleen. The frequency of Ki67^+^ cells and Eomes expression among naive and memory subpopulations are depicted in the right panels. Each dot represents an individual mouse. (**b**) Thirty days after injection, mice were analysed for the absolute number of VM and TM CD8^+^ T-cell subsets in the spleen. Each dot represents an individual mouse. Data are representative of three independent experiments. (**c**) WT, IFNAR^−/−^ and Eomes^fl/fl^CD4^cre^ (EomesΔT) mice were injected with polyI:C and analysed at the indicated time points for the frequency of VM CD8^+^ T cells in blood samples. One month after injection, mice were killed and the absolute numbers of VM CD8^+^ T cells in indicated strains or WT littermates are illustrated. For the upper panels, each dot represents the mean of 5–7 mice per group. For the bottom panels, each dot represents an individual mouse. For all graphs, bars represent mean±s.e.m. *P<0.05 **P<0.01 (non-parametric Mann–Whitney).

Our results clearly demonstrate a role of IRF9 in the homeostasis of VM cells in steady state and upon aging. Injection of polyI:C induced an important upregulation of Eomes expression in CD8^+^ T cells 16 h after injection (Fig. 2e). PolyI:C was previously shown to induce the expansion of CD8^+^ memory T cells in a type I IFN- and IL-15-dependent manner^25^,^41^. Three days after injection, both VM and TM CD8^+^ T-cell subsets expanded significantly (Fig. 5a). This was reflected by a strong increase in their proliferation rate (Fig. 5a). In contrast to *in vitro* mRNA data (Fig. 2c), showing transient induction of Eomes by IFNβ, we observed that *in vivo*, increased Eomes protein expression in naive or memory subsets was maintained 3 days after polyI:C injection (Fig. 5a). To study the long-term effects of this stimulation, we analysed VM and TM cell subsets 1 month after polyI:C injection. The expansion of memory CD8^+^ T cells was still observed at this later time point in the VM compartment but not in the TM compartment (Fig. 5b). These short- and long-term effects on VM cells were abrogated in IFNAR^−/−^ mice confirming the role of type I IFNs in this process (Fig. 5c). To address the role of Eomes in the effects of type I IFNs on the homeostasis of memory cells, we next used Eomes^fl/fl^CD4^Cre^ mice. PolyI:C-induced expansion of VM CD8^+^ T cells did not occur in these mice (Fig. 5c). Taken together, these experiments support the involvement of a type I IFN/Eomes axis in the homeostasis of VM cells.

### Effects of type I IFN signalling on Ag-specific CD8^+^ T cells

**Figure 6:**
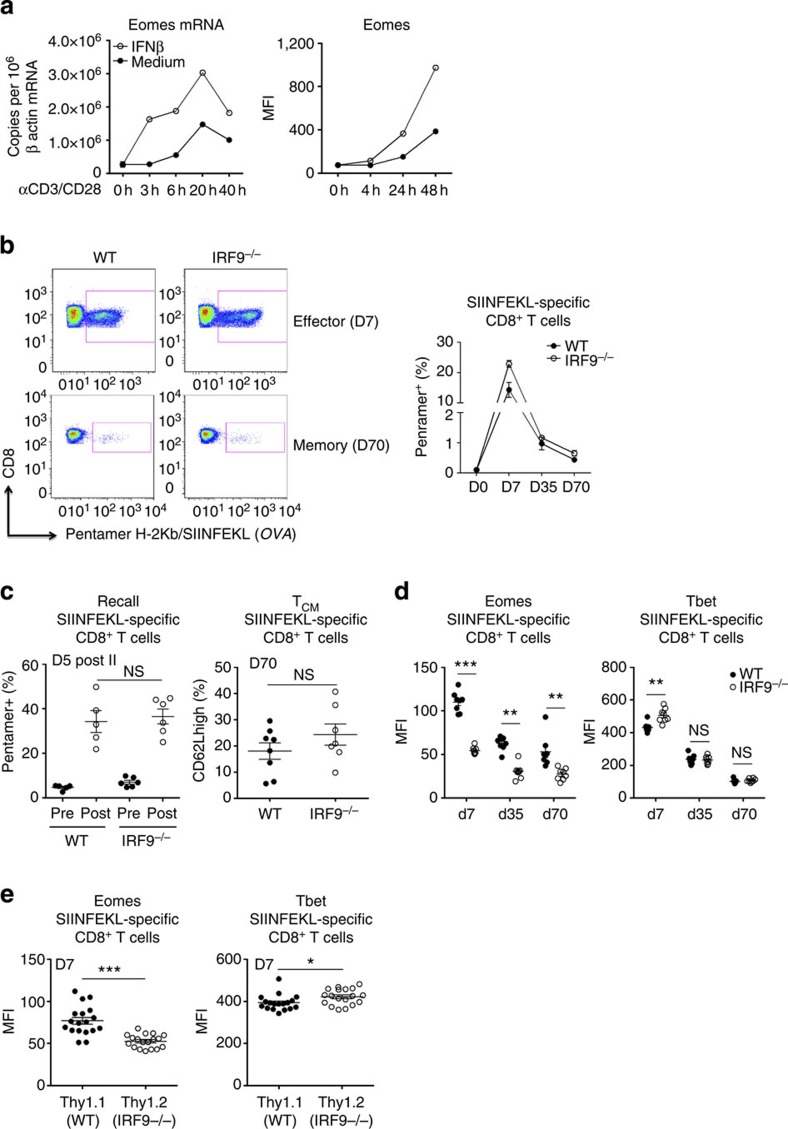
Effects of type I IFN signalling on Ag-specific CD8^+^ T cells. (**a**) Purified CD8^+^ T cells were activated by polyclonal stimulation (αCD3/28) *in vitro* with or without rIFNβ. Eomes expression is shown at the mRNA level by qRT–PCR (mean fluorescence intensity (MFI), left panel) and at the protein level by fluorescence-activated cell sorting (FACS) (right panel). (**b–d**) WT and IRF9^−/−^ mice were infected by LM deleted for *ActA* that expresses the ovalbumin antigen (Δ*actA* rLmOVA). (**b**) Blood samples were harvested at the indicated time after infection to monitor SIINFEKL-specific CD8^+^ T-cell responses. For this purpose, blood cells were stained with H-2Kb/SIINFEKL pentamers. Representative dot plots illustrate the frequency of Ag-specific CD8^+^ T cells in WT and IRF9^−/−^ mice. Graphs represent mean±s.e.m. of at least seven mice per group and are representative of three independent experiments (**c**). Mice were killed following 5 days of rLmOVA challenge at day 18 (recall phase) and at day 70 post infection (memory phase) and analysed, respectively, for the frequency of SIINFEKL-specific CD8^+^ T cells and the proportion of CD44^+^CD62L^+^ (central memory) cells. (**d**) At day 7 (effector phase) and days 35–70 (memory phase) of infection, the mice were killed and splenocytes were collected. Eomes and T-bet expression (MFI) within SIINFEKL-specific CD8^+^ T cells were determined by FACS staining. (**e**) Mixed bone marrow chimera were generated by transferring WT Thy1.1^+^ and IRF9^−/−^ Thy1.2^+^ myeloid progenitors into the irradiated RAG2^−/−^ host. These mice were inoculated with Δ*actA* rLmOVA and killed at day 7 post infection to analyse the expression of Eomes and T-bet among SIINFEKL-specific CD8^+^ T cells. Each dot represents an individual mouse. Mean±s.e.m. is shown. *P<0.05, **P<0.01 and ***P<0.001, NS: not significant (non-parametric Mann–Whitney or paired Wilcoxon test for mixed bone marrow chimera experiments).

Data obtained in steady state or polyI:C-treated mice suggest that type I IFN signalling contributes to the homeostasis of VM but not TM cells. To specifically address the role of IRF9 in antigen-specific CD8^+^ T cells, we first assessed the effect of type I IFN stimulation in the context of an *in vitro* polyclonal activation assay. Consistent with previous reports^42^, we observed that type I IFNs potentiated polyclonal-induced Eomes expression (Fig. 6a). We next evaluated the role of this signalling pathway upon antigenic challenge *in vivo*. For this purpose, we infected WT or IRF9^−/−^ animals with LM deleted for *ActA* that expresses the ovalbumin (OVA) antigen (Δ*actA* rLmOVA)^43^, and we analysed SIINFEKL-specific CD8^+^ T cells in the course of infection (Fig. 6b). In absence of IRF9, the frequency of OVA-specific CD8^+^ T cells in the effector phase was slightly increased (Fig. 6b). This could be related to the deleterious effects of type I IFN signalling in the course of *Listeria* infection^27^. The frequency of memory cells, their capacity to expand upon secondary challenge or the proportion of central memory CD8^+^ T cells were not affected in absence of IRF9 (Fig. 6c). We could demonstrate that Eomes expression was decreased in antigen-specific CD8^+^ T cells from IRF9^−/−^ mice during the effector phase (day 7 post infection) and the memory phase (days 35–70) (Fig. 6d). These results confirm the data obtained in total CD44^+^CD49d^hi^ cells (TM) under steady-state conditions (Fig. 3b). In contrast, T-bet expression in IRF9^−/−^ mice was slightly increased (effector phase) or unaffected (memory phase) (Fig. 6d). WT/IRF9 mixed bone marrow chimera experiments confirmed the T-cell intrinsic role of IRF9 in the induction of Eomes expression in OVA-specific CD8^+^ T cells upon Δ*actA* rLmOVA infection (Fig. 6e).

These results indicate that IRF9 contributes to Eomes expression in the course of antigenic activation and differentiation into antigen-specific memory CD8^+^ T cells. However, in sharp contrast to VM cells, the maintenance and function of conventional memory CD8^+^ T cells were not compromised in absence of IRF9 signalling, a finding that could be related to the partial contribution of Eomes to their maintenance or phenotype^15^.

### IRF9 regulates the homeostasis of innate CD8^+^ thymocytes

**Figure 7:**
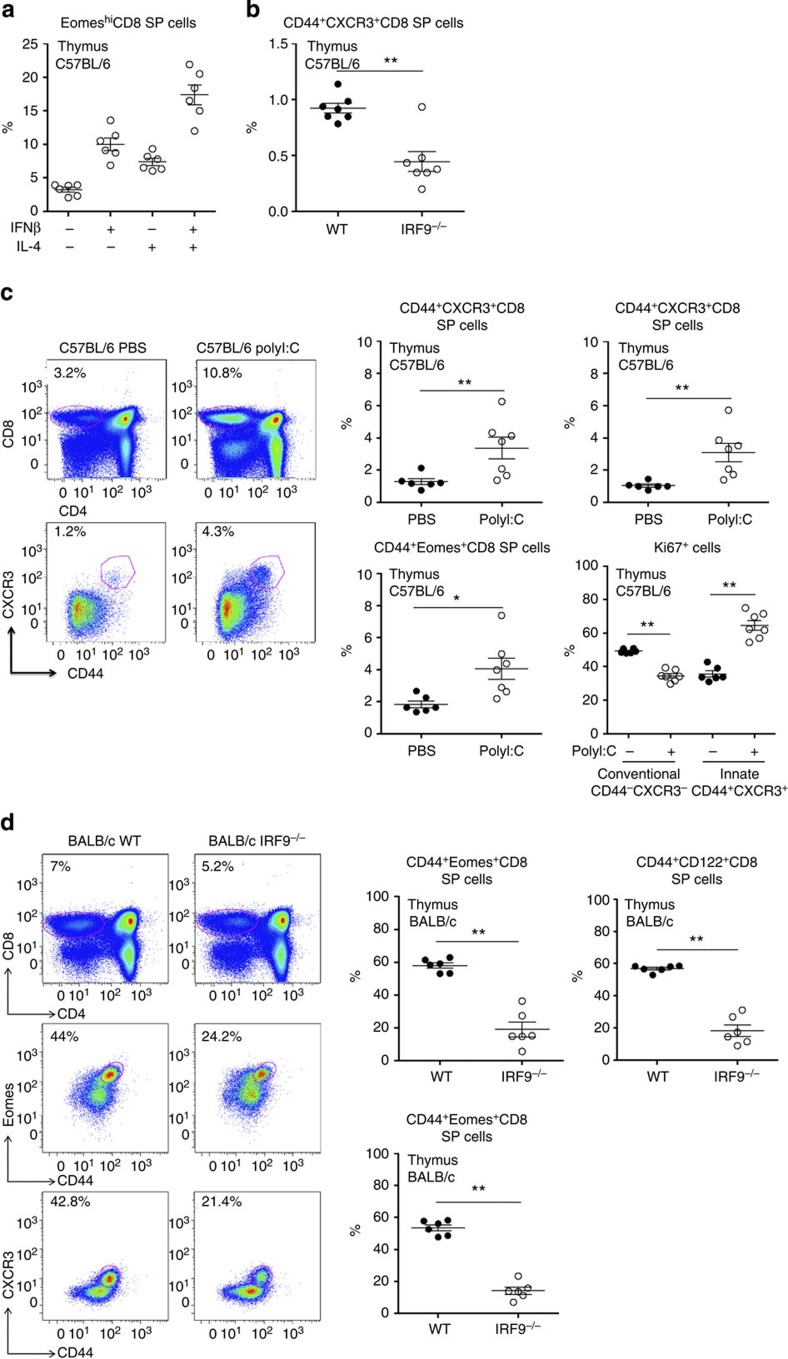
Type I IFNs regulate the homeostasis of innate CD8^+^ thymocytes. (**a**) Thymocytes from C57BL/6 mice were incubated in medium alone or stimulated with rIFNβ (100 U ml^−1^) and/or rIL-4 (20 ng ml^−1^). After 16 h, Eomes expression was assessed by flow cytometry among CD8 SP thymocytes. (**b**) The frequency of innate thymic CD8^+^ T cells (CD44^+^CXCR3^+^ CD8 SP) was analysed among CD8 SP thymocytes from C57BL/6 WT and IRF9^−/−^ mice. (**c**) PBS or polyI:C (200 μg per mouse) was injected intraperitoneally into C57BL/6 mice. After 3 days, mice were killed and thymocytes were isolated for flow cytometry analysis. The frequency of innate thymic CD8^+^ T cells (CD44^+^CXCR3^+^, CD44^+^CD122^+^ and CD44^+^Eomes^+^ CD8 SP) was analysed among CD8 SP thymocytes. The frequency of Ki67^+^ was assessed in conventional or innate CD8 SP thymocytes. (**d**) The frequency of innate thymic CD8^+^ T cells (CD44^+^CXCR3^+^, CD44^+^CD122^+^ and CD44^+^Eomes^+^ CD8 SP) was analysed among CD8 SP thymocytes of BALB/c WT and IRF9^−/−^ mice. For all the graphs, each dot represents an individual mouse. Bars represent mean±s.e.m. *P<0.05 and **P<0.01 (non-parametric Mann–Whitney).

Our results highlight the role of type I IFN/IRF9/Eomes pathway in the homeostasis of unconventional rather than conventional memory CD8^+^ T cells. Another memory-like CD8^+^ T-cell population, characterized by high expression of CD44, CXCR3, CD122 and Eomes, has been described in the thymus^4^,^12^. IL-4 is known to facilitate Eomes expression in CD8 SP thymocytes and the acquisition of this particular phenotype^19^,^35^,^44^. Indeed, we confirmed that incubation with rIL-4 induced Eomes expression in CD8 SP thymocytes (Fig. 7a). Consistent with our results in peripheral CD8^+^ T cells (Fig. 2a), rIFNβ alone or in combination with rIL-4 also induced Eomes expression in these experimental conditions (Fig. 7a). These results suggest that type I IFN signalling could participate in the development of innate thymic CD8^+^ T cells. Despite the low abundance of these cells in the thymi of C57BL/6 mice, we observed a significant reduction in the frequency of this population in IRF9^−/−^ mice under steady-state conditions as compared with WT animals (Fig. 7b). Type I IFNs are known to affect the survival of thymocytes^45^. However, 3 days after polyI:C injection, despite a strong decrease in the cellularity of the thymus, we observed a global rise in the proportion of CD8 SP cells in the thymus, suggesting that these cells might be relatively spared. Moreover, as observed in the periphery, we found an increase in the proportion of thymic innate cells among CD8 SP thymocytes in polyI:C-injected animals (Fig. 7c). PolyI:C induced a decrease in the proportion of Ki67^+^ classical CD44^−^CD8^+^ SP cells, which could reflect reduced proliferation rate or overall loss in CD4^+^CD8^+^ double-positive precursors. In sharp contrast, the proportion of Ki67^+^ cells among innate memory CD8 SP increased, indicating that along with Eomes expression, type I IFNs promote their expansion (Fig. 7c).

While innate CD8^+^ T cells are rare in the thymi of C57BL/6 mice, they were found to be abundant in BALB/c mice under steady-state conditions, as this population is dependent on IL-4-expressing NK T cells that are highly represented in this strain^4^,^19^. We therefore evaluated the proportion of innate thymic CD8 SP thymocytes in WT or IRF9^−/−^ BALB/c mice. This population was significantly reduced in IRF9^−/−^ mice as compared with their WT counterparts (Fig. 7d). Taken together, these results indicate that together with IL-4, type I IFNs contribute to the development of innate thymic CD8^+^ T cells.

## Discussion

Upon sensing microbial signals, APCs such as inflammatory monocytes, subcapsular macrophages or conventional dendritic cells produce cytokines that may directly or indirectly influence the function of CD8^+^ T cells^9^,^22^. Among these mediators, type I IFNs are known to regulate many aspects of T-cell functions^20^,^21^,^46^. Herein, we clearly demonstrate a direct action of type I IFNs and IRF9-dependent signals on *Eomes* gene activation in CD8^+^ T cells. This effect was observed in peripheral naive and memory CD8^+^ T cells and in CD8 SP thymocytes.

We show that type I IFNs favour the expansion of unconventional ‘VM' CD8^+^ T cells in an Eomes-dependent manner. This was found to be the case upon induction of type I IFNs in the context of polyI:C injection, and we observed that IRF9 was also required for the accumulation of this subset with age. How Eomes drives the acquisition of a memory-like phenotype is not clear. An important direct target of Eomes is CD122, a subunit shared by the IL-2 and IL-15 receptor^38^. Indeed, expression of T-bet and/or Eomes is required and sufficient to confer cellular responsiveness to IL-15 (ref. 38). This cytokine is critical for the maintenance of memory cells in general and the development of VM cells in particular^13^,^23^,^47^,^48^,^49^,^50^,^51^,^52^. Importantly, type I IFNs signalling within dendritic cells and/or inflammatory monocytes is also required for IL-15 production and efficient trans-presentation to CD8^+^ T cells^23^,^52^. Hence, both reduced responsiveness to homeostatic cytokines and inefficient IL-15 trans-presentation could be responsible for a lower proliferation rate and Bcl2 expression by IRF9^−/−^ VM cells^50^. Bone marrow chimera experiments clearly indicate that type I IFN signalling in CD8 T cells influences VM cell homeostasis. Taken together with previous works, we propose that type I IFNs directly upregulate Eomes expression in naive T cells leading to the induction of CD122 that confers responsiveness to IL-15 and subsequent conversion into VM cells. Type I IFNs might also be required to maintain high Eomes expression on VM cells, thereby favouring their survival and IL-15-driven expansion. Further work will be necessary to define the global transcriptional program imposed by Eomes in these cells. There is no direct evidence that upregulation of *Eomes* gene expression by type I IFNs are directly responsible for our observations. It is important to note that the effects of type I IFNs on VM CD8 T cells probably involve other pathways that have not been investigated in this work. For example, type I IFNs were shown to promote IL-15Rα expression in human T cells, resulting in enhanced IL-15 signalling^53^.

The function of these memory-like cells is still a matter of debate^6^,^9^,^11^,^33^,^36^. Like classical memory CD8^+^ T cells, VM cells are able to rapidly produce IFNγ during the first stage of an infection, in absence of antigenic recognition^9^. They respond to specific antigens with a better proliferation rate than true naive CD8^+^ T cells^6^. These cells could therefore contribute both to innate and adaptive immune responses. Furthermore, memory-like cells are present in large amount in the neonatal period and also accumulate with age, so they could play an important role in these periods of high susceptibility to infections^32^,^33^,^36^,^54^,^55^. It is tempting to speculate that high Eomes (and/or T-bet) expression is responsible for these innate properties. Indeed, in response to IL-12/IL-18 stimulation, IFNγ production was restricted to Eomes-positive CD8^+^ T cells. Furthermore, in absence of IRF9 within CD8^+^ T cells, the proportion of VM cells, their Eomes expression and their capacity to produce IFNγ were decreased.

IRF9 was also required for optimal Eomes expression in the context of antigen-driven differentiation into effector and memory cells *in vivo*. Despite these findings, the persistence or the phenotype of these ‘TM' cells under steady-state conditions, upon polyI:C injection or in the context of *Listeria* infection, was not consistently affected in absence of type I IFN signalling. We cannot exclude that IRF9-dependent induction of Eomes in antigen-experienced cells could contribute to their long-term maintenance in other contexts. However, this hypothesis is difficult to test in viral models such as lymphocytic choriomeningitis virus infection. Indeed, in contrast to *Listeria* infection, type I IFNs are critical for the induction of effector CD8^+^ T cells in these models, which precludes the analysis of their role in the memory phase^26^,^46^,^56^.

Another unconventional memory CD8^+^ T-cell population arises in the thymus. They differ from VM cells as this innate-like population is strongly dependent on IL-4 produced by PLZF-expressing NK T cells that are highly represented in BALB/c mice^4^,^19^. Of note, the thymus was shown to be an important source of type I IFN under physiological conditions^57^,^58^. Furthermore, we observed that rIFNβ strongly potentiated IL-4 driven Eomes expression, suggesting that type I IFNs could also influence the homeostasis of thymocytes. Indeed, we show that the proportion of Eomes^+^ innate CD8 SP thymocytes was significantly decreased in IRF9^−/−^ BALB/c mice. This result indicates that, in addition to IL-4, type I IFN signalling contributes to the differentiation of these memory-like cells.

In conclusion, we demonstrate that type I IFN signalling within CD8^+^ T cells directly regulates Eomes expression, a transcription factor associated with the acquisition of a memory-like phenotype and innate functions. Together with the role of type I IFNs on IL-15/IL-15R expression, we show that this mechanism contributes to the long-term homeostasis, fitness and function of VM CD8^+^ T cells in the periphery and the differentiation of innate memory cells in the thymus.

## Methods

### Mice

IRF9-deficient (IRF9^−/−^) mice on C57BL/6 or BALB/c backgrounds were obtained from the Riken BioResource Center (Ibaraki, Japan) with the approval of T. Taniguchi (University of Tokyo, Tokyo, Japan). RAG2^−/−^, CD3ɛ^−/−^, Thy1.1 and CD45.1 congenic mice on C57BL/6 background were obtained from the Jackson Laboratory. STAT1^−/−^ and IFNAR1^−/−^ mice on C57BL/6 background were kindly provided by D.E. Levy (New York University School of Medicine, NYC, USA) and Claude Libert (Department for Molecular Biomedical Research, VIB, Ghent, Belgium), respectively. These mice were housed and bred in our specific pathogen-free animal facility. Eomes^flox/flox^ mice were previously described^59^. These mice were further back-crossed onto C57BL/6 background for eight generations. To inactivate Eomes in T lymphocytes, mice were crossed with CD4-Cre animals. All experimental groups were matched for sex and age (6–10 weeks or as indicated in the legend of the figures). All animal studies were approved by the institutional Animal Care and local committee for animal welfare.

### Cell preparation and cultures

Total CD8^+^ T cells were purified by magnetic-activated cell sorting (CD8^+^ microbeads, mice; MACS Miltenyi Biotec or Dynabeads Untouched Mouse CD8 Cells Kit from Life Technologies). All cultures were performed in RPMI with 10% (vol/vol) FCS, 2 mM l-glutamine, 1 mM sodium pyruvate, 0.1 mM non-essential amino acids, 40 μM β-mercaptoethanol, 100 U ml^−1^ of penicillin and 100 U ml^−1^ of streptomycin (all from Lonza). Total CD8^+^ T cells were cultured with or without rIFNβ at 100 U ml^−1^ (PBL Interferon Source) and rIL-4 at 20 ng ml^−1^ (R&D Systems). In addition, we performed cocultures with rIFNβ and plate-bound anti-CD3 (5 μg ml^−1^; 145-2C11, BD biosciences) and soluble anti-CD28 (1 μg ml^−1^; 37.51, BD biosciences). In another set of experiments, we performed Thy1.1^+^/Thy1.2^+^ cocultures; Thy1.2^+^ (CD90.2^+^) CD8^+^ T cells purified from WT or IRF9^−/−^ mice were cultured with WT Thy1.1^+^ (CD90.1^+^) splenocytes for 16 h in the presence or absence of rIFNβ. In another panel of experiments, splenocytes were cultured for 16 h with a cocktail of rIL-12 (5 ng ml^−1^) and rIL-18 (10 ng ml^−1^, MBL) or medium alone.

### *In vivo* experiments

For adoptive transfer experiments using polyI:C, 3.10^6^ WT or IRF9^−/−^ CD8^+^ T cells (CD90.2^+^) were injected intravenously (i.v.) into congenic C57BL/6 recipient mice (CD90.1^+^). After 24 h, recipient mice were injected intraperitoneally with 200 μg of polyI:C (GE Health Care). After 24 h, recipient mice were killed and donor-derived T cells were identified by surface staining with anti-CD90.2 Pe-Cy7 (Biolegend, clone 30-H12m, 1/100) and anti-CD8 PerCP (BD biosciences, clone 53–6.7, 1/50).

For *in vivo* infections, WT and IRF9^−/−^ mice were inoculated i.v. with a recombinant attenuated strain of LM deleted for *ActA* that expresses the OVA antigen^43^ (Δ*actA* rLmOVA) or a recombinant strain of LM expressing ovalbumin (LM-OVA)^43^,^60^ (kindly provided by Dr Hao Shen, Department of Microbiology, University of Pennsylvania School of Medicine, Philadelphia).

Antigen-specific T-cell responses were monitored in blood and spleen using pentamer stainings (H-2Kb/SIINFEKL (*OVA*), Proimmune). To study innate CD8^+^ T-cell response in blood and spleen, animals were killed 16 h post injection of Δ*actA* rLmOVA i.v. (5 × 10^5^ colony-forming units per mouse). To evaluate the recall of the CD8^+^ T-cell response, mice were killed at day 5 following an injection of LM-OVA (1.10^6^ colony-forming units per mouse).

We also performed mixed bone marrow chimera by injecting either WT Thy1.1^+^ and IRF9^−/−^ Thy1.2^+^ or WT CD45.1^+^ and IFNAR^−/−^ CD45.2^+^ myeloid progenitors into lethally irradiated RAG2^−/−^ host recipient mice. When indicated, 8 weeks post engraftment, animals were inoculated with Δ*actA* rLmOVA and followed the same protocol as described previously.

### Cell surface and intracellular staining

Purified CD8^+^ T cells, splenocytes and thymocytes were first stained for surface antigens and then treated with Foxp3 staining buffer set according to the manufacturer's directions (eBioscience). Anti-Eomes AlexaFluor 647 or eFluor 660 (Dan11mag, 1/75), anti-T-bet PE (eBio4B10, 1/100) and anti-CD49d FITC or PE (R1-2, 1/50) antibodies were purchased from eBioscience. Anti-CD8 PercP (53–6.7, 1/50), anti-CXCR3 APC (CXCR3-173, 1/50), anti-CD4 Pe-Cy7 (RM4-5, 1/100), anti-CD62L PE (1/100) or V450 (1/50) (MEL-14), anti-Bcl2 PE (3F11, 1/25), anti-Ki67 FITC (B56, 1/25), anti-CD44 FITC or V450 (IM7, 1/50), anti-CD127 Pe-Cy7 (SB/199, 1/50), anti-CD122 FITC (TM-BETA1, 1/50), anti-NK1.1 FITC (PK136, 1/50), anti-CD90.2 Pe-Cy7 (53-2.1, 1/100) and anti-IFNγ APC or PB or PE (XMG1.21/50) were purchased from BD biosciences. Anti-CD3 Pe-Cy7 (2C11, 1/100) was purchased from Biolegend.

In some experiments, brefeldin A (5 μg ml^−1^, Sigma) was added in samples for 3 h at 37 °C before intracytoplasmic staining. Blood samples were directly stained for surface antigens and then treated with FACS lysing buffer (BD biosciences) as described in the product data sheet. All samples were fixed with 1% paraformaldehyde in PBS prior to their processing using a Cyan flow cytometer (Dako Cytomation).

### RNA purification and real-time RT–PCR

Total RNA from cells was extracted using a MagnaPure LC RNA-High Performance Isolation Kit (Roche Diagnostics). Reverse transcription (RT) and real-time PCR reactions were carried out using an RNA amplification kit (one-step procedure) on a Lightcycler 480 Real-Time PCR system (Roche Diagnostics). The sequences of primers and probes were: β-actin: 5′- TCCTGAGCGCAAGTACTCTGT -3′, 5′- CTGATCCACATCTGCTGGAAG -3′ and probe 5′- ATCGGTGGCTCCATCCTGGC -3′, Eomes: 5′- CCTTCACCTTCTCAGAGACACAGTT -3′, 5′- TCGATCTTTAGCTGGGTGATATCC -3′ and probe 5′- TCGCTGTGACGGCCTACCAAAACA -3′, T-bet: 5′- CAAGTTCAACCAGCACCAGA -3′, 5′- CCACATCCACAAACATCCTG -3′ and probe 5′- TCATCACTAAGCAAGGACGGCGA -3′.

### Chromatin immunoprecipitation

Purified CD8^+^ T cells were stimulated with rIFNβ (10^7^ cells per condition). Cells were then fixed for 10 min at room temperature with 1% formaldehyde, and glycine was added to a final concentration of 0.125 M. Cells were washed twice with ice-cold PBS, resuspended in lysis buffer and sonicated to obtain chromatin fragments that were 200–500 base pairs in length using a bioruptor device (Diagenode). Chromatin was then incubated overnight at 4 °C with monoclonal rabbit anti-H3K4me3 (Millipore), polyclonal rabbit anti-H3K9ac (Millipore), polyclonal rabbit anti-STAT1 (M-22, sc-592, Santa Cruz) or with rabbit polyclonal IgG (CS200581; Millipore) and protein G magnetic-activated beads (Active Motif). Beads were washed five times. Eluted samples were incubated with NaCl (final concentration: 200 mM) for 4 h at 65 °C. Samples were treated with RNAse and Proteinase K for 1 h at 45 °C and DNA was then purified using the QIAquick kit according to the manufacturer's instructions (Qiagen). Quantitative PCR was performed with primers encompassing the proximal promoter region of *Eomes*. The sequences of primers for *Eomes* were: 5′- AAAGAAACACCAAACCAGCA -3′, 5′- GGGACTTTGCTATTGGCTGT -3′ and probe 5′- CGCAGGCGACCCGATCCAATTA -3′. As a positive control for histone modifications, we used primers located in the *Gapdh* proximal promoter region: 5′- CCACCATCCGGGTTCCTAT -3′, 5′- GCGATTTTCACCTGGCACT -3′ and probe: 5′- CTCCTCCCTGTTCCAGAGACGGC -3′. As a negative control, we used a region located +14.6 kb downstream of *Eomes* transcriptional start site: 5′- GCGCACACACACACACATAC -3′, 5′- AAATGGCAGGTTTCTTTACCC -3′ and probe: 5′- CATCTCACTAGACCTTGAGTCAGTCCTCTCTCTC -3′.

### Statistical analysis

Statistical analysis was performed using a non-parametric Mann–Whitney or paired Wilcoxon test when appropriate.

## Author contributions

V.M., S.T. and M.N. conducted most of the experiments. D.T., A.A. and A.K. contributed to some experiments. C.-A.M. and W.H.K. provided critical reagents. V.M. and S.T. analysed the data. V.F. and O.L. provided input for research design and interpretation. V.M. and S.G. designed the experiments and wrote the manuscript.

## Additional information

**How to cite this article**: Martinet, V. *et al*. Type I interferons regulate eomesodermin expression and the development of unconventional memory CD8^+^ T cells. *Nat. Commun.* 6:7089 doi: 10.1038/ncomms8089 (2015).
